# Oxidative Damage in Human Periodontal Ligament Fibroblast (hPLF) after Methylmercury Exposure

**DOI:** 10.1155/2019/8470857

**Published:** 2019-11-22

**Authors:** Lygia S. Nogueira, Carolina P. Vasconcelos, Geovanni Pereira Mitre, Maria Sueli da Silva Kataoka, Marcelo O. Lima, Edivaldo H. C. de Oliveira, Rafael R. Lima

**Affiliations:** ^1^Universidade Federal do Pará, Laboratório de Biologia Estrutural e Funcional, Belém, Pará, Brazil; ^2^Instituto Evandro Chagas, Laboratório de Citogenética e Cultura de Tecidos-SAMAM, Ananindeua, Pará, Brazil; ^3^Universidade Federal do Pará, Laboratório de Cultura Celular, Belém, Brazil; ^4^Instituto Evandro Chagas, Laboratório de Toxicologia-SAMAM, Ananindeua, Pará, Brazil; ^5^Universidade Federal do Pará, Instituto de Ciências Exatas e Naturais, Brazil

## Abstract

Human exposure to mercury (Hg) is primary associated with its organic form, methylmercury (MeHg), through the ingestion of contaminated seafood. However, Hg contamination is also positively correlated with the number of dental restorations, total surface of amalgam, and organic mercury concentration in the saliva. Among the cells existing in the oral cavity, human periodontal ligament fibroblast (hPLF) cells are important cells responsible for the production of matrix and extracellular collagen, besides sustentation, renewal, repair, and tissue regeneration. In this way, the present study is aimed at investigating the potential oxidative effects caused by MeHg on hPLF. Firstly, we analyzed the cytotoxic effects of MeHg (general metabolism status, cell viability, and mercury accumulation) followed by the parameters related to oxidative stress (total antioxidant capacity, GSH levels, and DNA damage). Our results demonstrated that MeHg toxicity increased in accordance with the rise of MeHg concentration in the exposure solutions (1-7 *μ*M) causing 100% of cell death at 7 *μ*M MeHg exposure. The general metabolism status was firstly affected by 2 *μ*M MeHg exposure (43.8 ± 1.7%), while a significant decrease of cell viability has arisen significantly only at 3 *μ*M MeHg exposure (68.7 ± 1.4%). The ratio among these two analyses (named fold change) demonstrated viable hPLF with compromised cellular machinery along with the range of MeHg exposure. Subsequently, two distinct MeHg concentrations (0.3 and 3 *μ*M) were chosen based on LC50 value (4.2 *μ*M). hPLF exposed to these two MeHg concentrations showed an intracellular Hg accumulation as a linear-type saturation curve indicating that metal accumulated diffusively in the cells, typical for metal organic forms such as methyl. The levels of total GSH decreased 50% at exposure to 3 *μ*M MeHg when compared to control. Finally, no alteration in the DNA integrity was observed at 0.3 *μ*M MeHg exposure, but 3 *μ*M MeHg caused significant damage. In conclusion, it was observed that MeHg exposure affected the general metabolism status of hPLF with no necessary decrease on the cell death. Additionally, although the oxidative imbalance in the hPLF was confirmed only at 3 *μ*M MeHg through the increase of total GSH level and DNA damage, the lower concentration of MeHg used (0.3 *μ*M) requires attention since the intracellular mercury accumulation may be toxic at chronic exposures.

## 1. Introduction

Considered one of the top ten chemicals or groups of chemicals of major public health concern by the World Health Organization (WHO), mercury is released in thousands of tons into the environment mainly through uncontrolled gold-mining activities. Increased Hg levels are reported in water, sediments, and fishes [1, 2]. MeHg is a well-known neurotoxin and has been shown to disrupt the function of multiple organs throughout the human body [3].

The main aspect studied on mercury exposure is the oxidative stress scenery. A variety of *in vitro* and *in vivo* models have shown that MeHg binds to total glutathione (GSH). This protein is the substrate for glutathione S-transferase (GST) and plays a key role in cellular detoxification of xenobiotics and in excessive production of oxygen species. The decreased level of total GSH or the ratio between GSH/GSSG results in oxidative stress and evidences an important molecular mechanism in MeHg-induced toxicity [4, 5]. Related to mercury exposure, oxidative stress is also associated with mitochondrial dysfunction [6] and alterations on membrane permeability and macromolecule structure (DNA, protein, and lipids), due to their high affinity for sulphydryl groups and thiols [7].

Concerning human exposure to mercury, it is primarily associated particularly with the consumption of contaminated fish and other seafood that turns MeHg the most toxic form of this metal [8]. Additionally, it is important to consider that levels of mercury in the blood also have a positive correlation with the number of dental restorations [9], the total surface of amalgam, and organic mercury concentration in the saliva [10]. It is noteworthy that although mercury is found in metallic form in restorations, there are commensal bacteria or normal microflora found in the mucosal surfaces of oral cavity, which are involved in the methylation of mercury, turning it into the most toxic form of exposure [11, 12].

Among the different types of cells in the oral cavity, human periodontal ligament fibroblast (hPLF) cells are the most numerous population and responsible for different functions to maintain the periodontal homeostasis. These cells produce and secrete extracellular matrix components having the most production of collagen [13, 14]. Besides that, hPLF may also produce mineralized tissue, showing higher alkaline phosphatase activity and being consider an essential cell to play a role in the remodeling of alveolar bone [15]. Cells of the periodontal ligament also participate actively in immune and inflammatory events in periodontal diseases producing cytokine and chemokines [16] and have high active metabolism [17] probably due to their remarkable capacity for renewal and repair of the periodontal ligament; consequently, the effects caused by MeHg exposure on their metabolism probably implicate directly to their function in the oral cavity. These facts turn this cell type an essential and ideal model for MeHg research for oral cavity.

Based on this, the present study is aimed at investigating the effects of the exposure to MeHg in hPLF from toxicological and oxidative stress perspective. Firstly, *in vitro* experiments were performed to evaluate the effects of a range of MeHg concentrations (1-7 *μ*M) in hPLF general metabolism status and viability. Based on cell viability results, the lethal concentration for 50% of the population (LC50) was calculated by Probit method and two different MeHg concentrations (0.3 and 3 *μ*M) were chosen to proceed the experiments. Further, exposed hPLFs were assessed by intracellular mercury accumulation parameters of oxidative stress (GSH levels and DNA damage).

## 2. Material and Methods

### 2.1. Cell Culture

hPLFs were cultured in Dulbecco's modified Eagle's medium (DMEM) and Ham's F-12 nutrient medium (1 : 1), supplemented with 10% foetal bovine serum (FBS), 100 U/mL penicillin, and 100 *μ*g/mL streptomycin, incubated at 37°C in a 5% CO_2_. The medium was changed every 48 h. When cells became fully confluent, they were passaged using 0.25% trypsin solutions and seeded in new flasks. Passages until 15 were used in our experiments. This cell population was confirmed as human periodontal fibroblast cells by indirect immunofluorescence staining target antigens, the vimentin and fibronectin proteins.

### 2.2. General Metabolism Status

General metabolism status was measured using the MTT protocol [18]. For this, hPLFs were seeded in a 96-well plate at concentration of 1 × 10^4^ cell/well and exposed to a medium containing different concentrations of MeHg, 1, 2, 3, 4, 5, 6, and 7 *μ*M, with no FBS supplementation. Control group was maintained in fresh culture medium. After 24 h exposure, the medium was removed and replaced by new culture medium containing MTT (500 *μ*g/mL) and incubated for 2 hours. In the end, the medium was removed and 100 *μ*L of DMSO was added to dissolve the formazan crystals. Absorbance was recorded at 550 nm using GloMax®-Multi Detection System (Promega). Results were expressed in percentage of the control (%).

### 2.3. Cell Viability and LC50

hPLFs were seeded in a concentration of 1 × 10^4^ cells/well and cultured in fresh medium for 24 h. After this, the medium was replaced for a new medium containing MeHg in the same concentrations used for MTT, with no FBS supplementation. After the exposure time, the medium was removed and hPLFs were washed with EDTA solution (10 mM) to remove possible loosely bound mercury of the cell surface. Following that, cells were detached using trypsin and centrifuged (2300*g*, 5 minutes). Pellets containing the hPLF were resuspended in fresh culture medium and counted under a light microscope (200x magnifications). Cell viability (% of viable cells from the total number of cells) was determined using Trypan Blue (0.04%) exclusion assay. The results were expressed in percentage (%) and used to determine the lethal concentration for 50% of the population (LC50) by Probit analysis. Based on LC50 results, two MeHg concentrations that represent 7 and 70% of LC50 were chosen to perform the following analyses: 0.3 and 3 *μ*M, respectively. The lower concentration (0.3 *μ*M or ~60 *μ*g/L) represents values observed in humans exposed at Brazilian Amazon region communities while 3 *μ*M (or ~600 *μ*g/L) is considerably toxic.

### 2.4. Ration between General Metabolism Status and Cell Viability

Cells may vary their metabolism due to exposure to physical or chemical agents, whether or not related to different doses or concentrations of the compounds. However, variations in MTT assay values may not reflect these alterations because it is not directly related to a possible decrease or increase of the number of viable cells after experimentation [19]. Thus, in our study, it will analyze the ratio between the general metabolism status (MTT assay) and the cell viability at correspondent treatment. As a result, this analysis will enable us to demonstrate an increase or decrease in cell metabolic status of hPLF related to the different MeHg exposures. The results were expressed as fold change.

### 2.5. Intracellular Hg Concentration

hPLFs were detached from 24-well microplates after exposure to 0.3 and 3 *μ*M MeHg and centrifuged (2300*g*, 5 minutes), and the pellets were dried overnight (37°C). Following that, samples were digested with nitric acid (Merck, St. Louis, MO, USA) and diluted with Milli-Q water. The total Hg concentration (THg) in the digested samples was analyzed by cold vapour atomic absorption (Automatic Analyzer, HG-20, Sanso Company), as previously described by Akagi et al. [20]. Hg content in hPLF cells was expressed as *μ*M THg/10^5^ cells, considering the amount of Hg measured in the cell.

### 2.6. GSH Levels

Levels of total glutathione in the reduced form (GSH) were analyzed using GSH/GSSG-Glo™ Assay (Promega, Madison, WI, USA) according to the manufacturer's instruction. After 24 h MeHg exposure, the medium was removed and cell lysis was performed using total glutathione reagent for 5 minutes. Following that, the lysate was incubated with luciferin generation reagent. After 30 minutes, luciferin detection reagent was added to each well and solution equilibrated for 15 minutes at room temperature. Luminescence was read using GloMax®-Multi Detection System (Promega) and data expressed as *μ*M GSH/viable cells.

### 2.7. Comet Assay

DNA damage was analyzed using the single-cell gel electrophoresis (SCGE) alkaline comet assay, based on the protocol described by Sing and Stephens [21]. hPLFs exposed to 0.3 and 3 *μ*M were detached after 24 h MeHg exposure, and the formed pellet was resuspended into 300 *μ*L of new cell culture medium. An aliquot (20 *μ*L) was homogenated with 120 *μ*L of low-melting agarose and added to the slides pretreated with agarose layer. After drying, slides were incubated in lyse solution (in M: 2.5 NaCl, 0.1 EDTA, 0.01 Tris, 1% Triton X-100) and maintained overnight at 4°C. Following that, slides were placed into the electrophoresis solution (in mM: 300 NaOH, 1 EDTA; pH 13) for 20 minutes for the unwinding of the DNA. Electrophoresis was performed for 20 minutes at 30 V (1 V/cm) and 300 mA. The last steps were to neutralize the slides using 0.4 M Tris buffer (pH 7.5), stain them with DAPI (Enzo Life Sciences, NY, USA), and analyze them using a fluorescence microscopy (Zeiss Imager Z2, connected to the software Axiovison 4.8, Zeiss, Alemanha). One hundred cells per sample were automatically analyzed through Komet Software®. DNA damage was expressed as the length of the comet tail in percentage.

### 2.8. Data Presentation and Statistical Analyses

Experiments were performed using three different passage numbers (*n* = 3). Therefore, each value represents the mean of these replicates and they are expressed as the mean ± standard error. Comparisons among treatments were performed using factorial analysis of variance (ANOVA) followed by the Tukey test. ANOVA assumptions (data normality and homogeneity of variances) were previously verified. Data were mathematically transformed when necessary. In all cases, the significance level adopted was 95% (*α* = 0.05). Statistical analyses were performed using the software SigmaPlot 11.0 (Systat Soft, Germany).

## 3. Results

### 3.1. MeHg Toxicity

**Figure 1:**
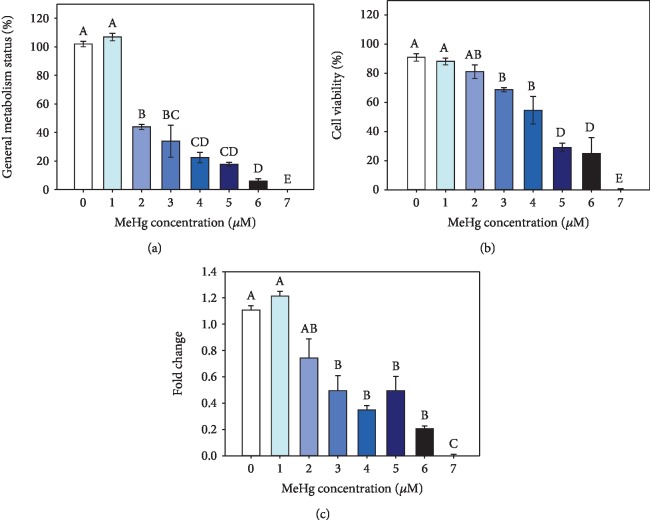
General metabolism status (a), cell viability (b), and ratio between metabolism status and cell viability (c) in hPLF exposed the range of 1 to 7 *μ*M MeHg. It was observed 100% of dead hPLF cells after 7 *μ*M MeHg exposure. Data are expressed as mean ± standard error (*n* = 3). Means sharing the same letters are not statistically significant.

General metabolism status in hPLF exposed to 1 *μ*M MeHg was similar to control but dropped significantly at 2 *μ*M MeHg exposure (43.8 ± 1.7%), declining until 6 *μ*M MeHg (5.9 ± 1.7%) and being not observed at 7 *μ*M MeHg treatment (Figure 1(a)).

Cell viability decreased significantly in the hPLF exposed to 3 *μ*M treatment (68.7 ± 1.4%). Exposure to 4 *μ*M MeHg had no statistical difference (54.5 ± 9.3%) compared to 3 *μ*M MeHg, while 5 and 6 *μ*M MeHg decreased significantly to 29.0 ± 2.9% and 24.6 ± 10.8%, respectively. No viable hPLFs were observed after exposure to 7 *μ*M MeHg (Figure 1(b)). Using cell viability results, the lethal concentration of 50% of population (LC50) was calculated and represented 4.2 *μ*M MeHg. Based on that, concentrations of 7 and 70% of the LC50 were chosen to perform the following analyses: 0.3 and 3 *μ*M, respectively.

The fold change calculated by the ratio between general metabolism status and cell viability demonstrated a significant decrease at 2 *μ*M MeHg exposure and remained similar until 6 *μ*M (Figure 1(c)) which implies a nondose response.

**Figure 2:**
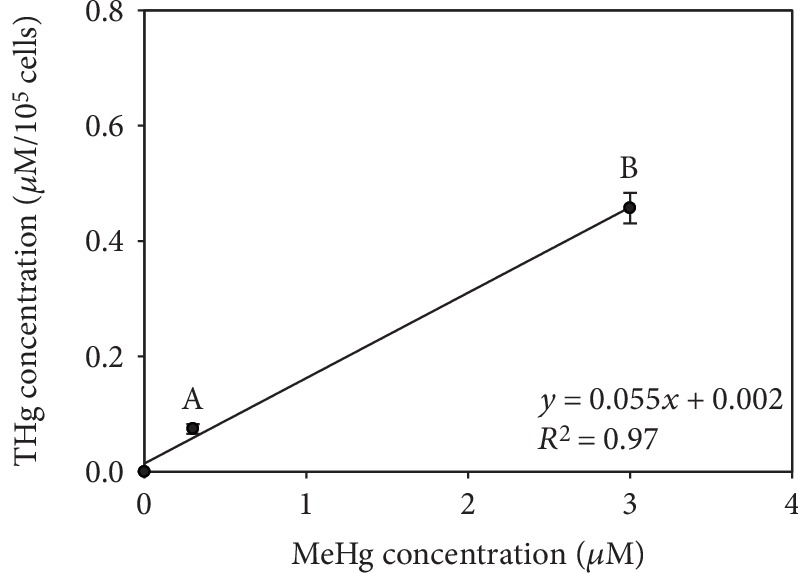
Total intracellular mercury concentration (THg) in hPLF exposed to 0.3 and 3 *μ*M MeHg for 24 h. Data are expressed as mean ± standard error (*n* = 3). Different letters indicate significantly different mean values among treatments.

Intracellular total Hg (THg) concentration in hPLF exposed to 0.3 and 3 *μ*M MeHg increased significantly to 0.074 ± 0.008 *μ*M and 0.457 ± 0.026 *μ*M, respectively. As expected, intracellular Hg was not observed in the control cells (Figure 2).

### 3.2. Oxidative Stress Parameters

**Figure 3:**
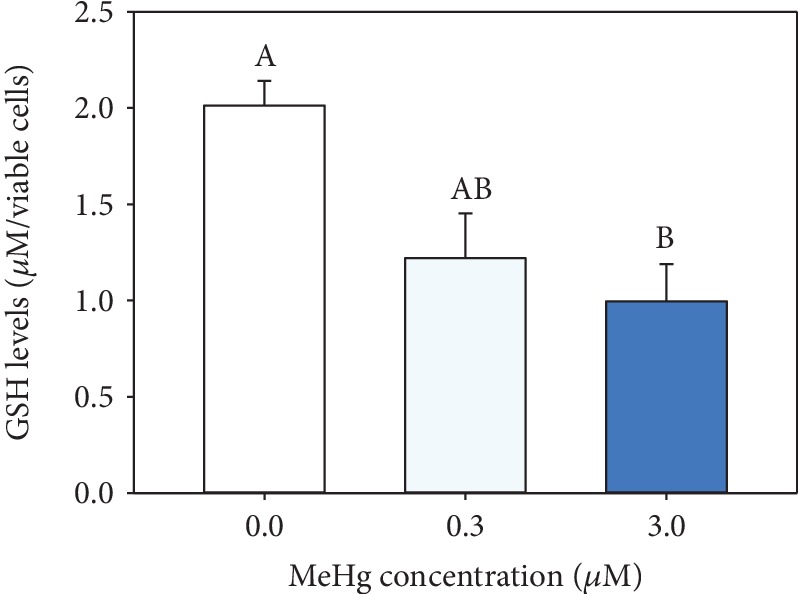
GSH levels in hPLF exposed to 0.3 and 3 *μ*M MeHg for 24 h. Data are expressed as mean ± standard error (*n* = 3). Means sharing the same letters are not statistically significant.

**Figure 4:**
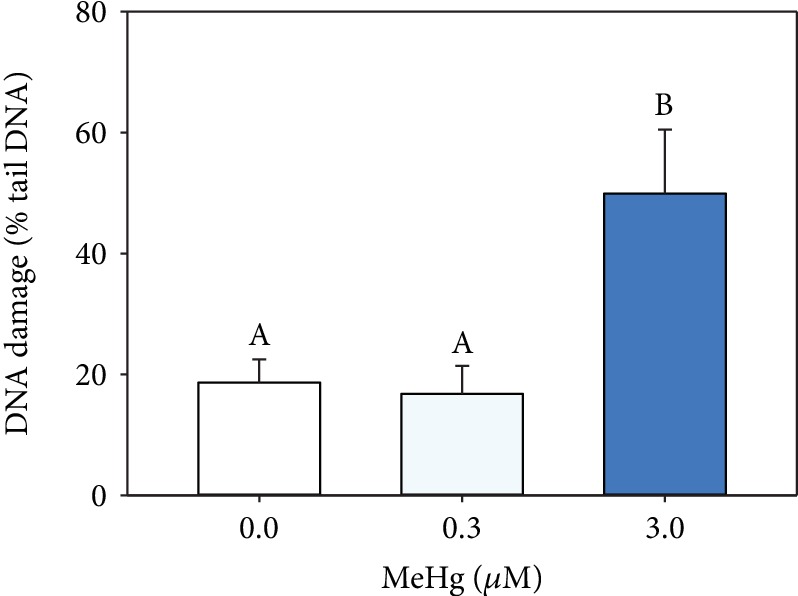
Percentage of DNA damage in the tail of hPLF kept under control (0 *μ*M MeHg) and exposed to 0.3 and 3 *μ*M MeHg for 24 h. Data are expressed as mean ± standard error (*n* = 3). Different letters indicate significantly different mean values among treatments.

The total glutathione (GSH) levels at hPLF had a significant decrease in the higher MeHg exposure (0.9 ± 0.1 *μ*M/viable cells) comparing to control (2.0 ± 0.12 *μ*M/viable cells) and 0.3 *μ*M MeHg exposure (1.2 ± 0.2 *μ*M/viable cells; Figure 3). Decreasing levels of total GSH is considered biomarker of oxidative stress, and it is confirmed in hPLF by a significant increase of DNA damage at 3 *μ*M MeHg (49.9 ± 10.6%) when compared to control and 0.3 *μ*M MeHg (18.6 ± 3.8% and 16.7 ± 4.6%, respectively; Figure 4).

## 4. Discussion

For the first time in the literature, *in vitro* experiments using hPLF were performed to evaluate the effects of MeHg exposure. In our study, these effects were primarily associated with oxidative stress parameters through the decreased level of total GSH and occurrence of DNA damage. However, hPLF general metabolism status was affected with no necessary changes on their cell viability. These combined results indicate an impairment of their cellular functions and consequently alterations in the periodontal homeostasis.

To initiate the evaluation on MeHg effects on hPLF, we firstly performed experiments using concentrations that ranged from 1 to 7 *μ*M and analyzed general metabolism status and cell viability. As expected, MeHg toxicity increased in parallel to the increase of metal concentration in the exposure solutions, causing 100% of not viable cells at 7 *μ*M MeHg. hPLF sensitivity is also observed after these cells were exposed to Cu, Ni, and Zn. However, San Miguel and co-authors [22] used higher concentrations and different times of exposure when compared to our study. Metal concentrations ranged from 30 to 40 *μ*M Cu or Zn and 1 to 2 mM Ni, and the exposure occurred for 60 minutes.

Interestingly, in the present study, hPLF cell viability decreased at 3 *μ*M MeHg exposure, but the general metabolism status dropped significantly at 2 *μ*M MeHg exposure. It is important to note that the MTT assay used in our study for general metabolism status evaluation is usually considered an appropriate indicator of mitochondrial function or directly related to the number of living cells [23, 24]. However, Stockert and co-authors [25] using intracellular fluorescent markers confirmed the biochemical evidences that MTT conversion occurs mainly in the cytoplasm by nicotinamide adenine dinucleotide coenzyme (NADH) and dehydrogenases associated with the endoplasmic reticulum [26, 27], lysosome vesicles [28], and plasma membrane [28]. Thus, the use of MTT assay as a direct measure of mitochondrial activity or living cells would be a highly indirect method [19]. Using this new approach for MTT assay, it was possible to observe through the fold change analysis that compromised hPLF cellular machinery is observed from the 2 *μ*M MeHg treatment and remains with no differences along the range of MeHg exposure, which implies in a nondose response.

From the measurements of cell viability, we were able to calculate the LC50 of hPLF that represents 4.2 *μ*M MeHg (or 842.5 *μ*g/L). Unfortunately, it was not possible to compare our LC50 results directly to previously published studies with other cell types, since they were calculated based on MTT assay, in spite of the relevant restrictions already demonstrated concerning the use of this method as an indicator of cell viability [19]. However, it is possible to mention that LC50 calculated in this study is extremely high when compared to mercury concentration found in human blood. In Brazilian Amazon fishing communities, the total mercury concentration in the blood is about 27 *μ*g/L (0.13 *μ*M) but some individuals have values above the average, such as 141 *μ*g/L (6.3 *μ*M) [29]. As mentioned in Material and Methods, to proceed the experiments evaluating MeHg effects on hPLF, we opted to use two different concentrations, 0.3 and 3 *μ*M.

hPLF exposed to 0.3 and 3 *μ*M of MeHg showed a linear increase of intracellular Hg accumulation. Although the experiments were performed using only two MeHg concentrations, linear-type kinetics observed in the accumulation reflects a diffusive accumulation of this metal from the extracellular medium. The presence of organic grouping methyl associated with Hg gives this metal this diffuse ability through biological/cellular membranes due to its lipophilic characteristic [30].

The presence of intracellular mercury is recognized to cause oxidative stress *in vivo* and *in vitro* studies [31]. GSH is the primary defense against the excessive generation of harmful ROS [4] by the presence of sulfhydryl group which serves as an antioxidant [32]. In this way, it was performed a specific GSH assay on hPLF exposed to MeHg. The depletion of total GSH in hPLF exposed to 3 *μ*M MeHg is explained by the interaction with intracellular thiols being the main target of MeHg. However, the mechanism of MeHg toxicity in hPLFs was different from those observed in glioblastoma cells [5]. No changes in the GSH levels were observed in exposed glioblastoma cells exposed to 1 *μ*M MeHg taking place a significant increase of GSSH levels (12-fold). The reduction of total glutathione level (GSH) is a confirmed endpoint of the misbalance between the production of reactive oxygen species and antioxidant defenses, which results in oxidative stress.

In the present study, another evidence of oxidative stress in exposed hPLF was the significant DNA damage at 3 *μ*M MeHg. Once accumulated, mercury is able to produce reactive oxygen species that react directly with DNA or induce conformational changes in DNA repair enzymes and protein of microtubules [33]. It is important to note that DNA damage in hPLF depends on MeHg concentration once cells exposed to 0.3 *μ*M MeHg did not differ from control. Comet assay applied in our study is a usual technique to evaluate the induced effects of metals released from orthodontic appliances on buccal cells [34, 35] and gingival fibroblasts [36]. It is possible to detect DNA single- and double-strand breaks, alkali-labile sites (ALS), DNA-DNA/DNA-protein cross-linking, and SSB associated with complete excision repair sites. Single- and double-strand breaks may cause apoptosis through inactivating key genes or leading chromosomal aberrations [37, 38]. In hPLF exposed to MeHg, the typical nucleus of apoptotic cells was not observed through comet assay analysis in the higher MeHg concentration. However, Contreras and coauthors [37] expose gingival fibroblast cells to Ni and observed apoptosis markers, such as DNA fragmentation and caspase-3 activation, which are characteristic of apoptosis. Additionally, Cu and Ni reduced significantly the DNA synthesis in gingival fibroblasts and hPLF [39] and DNA damage in oral mucosa cells [40]. Thus, although we did not observe hPLF in apoptotic process via comet assay, we strongly recommend further analyses to evaluate specifically apoptotic pathway in hPLF exposed to MeHg.

**Figure 5:**
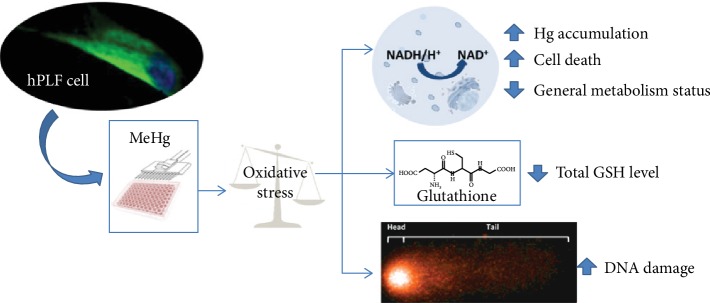
Graphical abstract with description of the main results found in this article.

Taken together, the use of the fold change analysis revealed the presence of viable hPLF along the range of metal exposures with compromised cellular machinery, arising especially between treatments of 2 and 4 *μ*M MeHg. Although these concentrations are not relevant for human mercury accumulation, the reduction on general metabolism status affects hPLF functions once oral cavity diseases may be related to other systemic problems. Furthermore, it is important to note that despite the lower MeHg concentration (0.3 *μ*M) did not exert any negative effect in the analyzed parameters, hPLF accumulated Hg which may implicate in different consequences if these cells were exposed to chronic manner. The main results found in this research are summarized in Figure 5.

## Data Availability

In our manuscript entitled “Oxidative damage in human periodontal ligament fibroblast (hPLF) after methylmercury exposure”, all data used to support the conclusions are presented within the manuscript. Therefore, no data from the repository were used to support this study.
